# Neuroplasticity in Parkinson’s disease

**DOI:** 10.1007/s00702-024-02813-y

**Published:** 2024-08-05

**Authors:** Bogdan Ovidiu Popescu, Lucia Batzu, Pedro J. Garcia Ruiz, Delia Tulbă, Elena Moro, Patrick Santens

**Affiliations:** 1 Department of Clinical Neurosciences, ‘Carol Davila’ University of Medicine and Pharmacy Bucharest, Bucharest, Romania; 2 Laboratory of Cell Biology, Neurosciences and Experimental Myology, ‘Victor Babeș’ National Institute of Pathology, Bucharest, Romania; 3 Department of Basic and Clinical Neuroscience, Institute of Psychiatry, Psychology and Neuroscience, The Maurice Wohl Clinical Neuroscience Institute, King’s College London, London, UK; 4 Parkinson’s Foundation Centre of Excellence, King’s College Hospital, London, UK; 5 Department of Neurology, Fundacion Jimenez Diaz, Madrid, Spain; 6 Division of Neurology, Centre Hospitalier Universitaire de Grenoble, Grenoble Alpes University, Grenoble Institute of Neuroscience, INSERM U1216, Grenoble, France; 7 Department of Neurology, University Hospital Ghent, Ghent, Belgium; 8 Faculty of Medicine and Health Sciences, Ghent University, Ghent, Belgium

**Keywords:** Parkinson’s disease, Neuroplasticity, Biomarkers, Dopamine, NMDA, AMPA, BDNF

## Abstract

Parkinson’s disease (PD) is the second most frequent neurodegenerative disorder, affecting millions of people and rapidly increasing over the last decades. Even though there is no intervention yet to stop the neurodegenerative pathology, many efficient treatment methods are available, including for patients with advanced PD. Neuroplasticity is a fundamental property of the human brain to adapt both to external changes and internal insults and pathological processes. In this paper we examine the current knowledge and concepts concerning changes at network level, cellular level and molecular level as parts of the neuroplastic response to protein aggregation pathology, synapse loss and neuronal loss in PD. We analyse the beneficial, compensatory effects, such as augmentation of nigral neurons efficacy, as well as negative, maladaptive effects, such as levodopa-induced dyskinesia. Effects of physical activity and different treatments on neuroplasticity are considered and the opportunity of biomarkers identification and use is discussed.

## Introduction

The quality of our brain activity, measured by functional performance, depends both on the number of neurons (*mass effect*) and the connections between them (*relationship effect*), enabling complex neurocircuitry. Complexity of behaviour is always possible due to the complexity of the underlying brain networks. Brain aging and neurodegeneration, associated with an overall decrease of brain connectivity to different extents and timing for each individual, might be seen as a reversal of brain development, when brain connectivity is increased. Aging neurons can be seen as aging humans, more fragile, and vulnerable, but still able to function and communicate. This review paper aims to look at neuroplasticity in an important neurodegenerative disorder, Parkinson’s disease (PD), shedding light on the mechanisms of communication adaptation under conditions of aging and death in the neuronal world of human brain.

### Neuroplasticity – definition and concept

Neuroplasticity is a fundamental property of the human brain that enables it to learn from and adapt to various experiences. This property was described for the first time by Ramon Y Cajal, in 1984, in the *Croonian lecture*: ‘the cerebral cortex is similar to a garden filled with trees, the pyramidal cells, which, thanks to intelligent culture, can multiply their branches, sending their roots deeper and producing more and more varied and exquisite flowers and fruits.’ (Jones 1994). The brain is in permanent change throughout life, continuing to reorganize pathways and create new connections (Byrne et al. 1990). Neuroplasticity does occur at various levels ranging from molecular alterations (e.g. protein expression, receptors externalisation) to cellular changes due to learning, to large scale cortical remapping in response to injury. The ability of the brain to rewire itself holds significant implications for developmental sciences, neurological recovery and medical rehabilitation (Seng et al. 2022). In the field of neurodegeneration, it is particularly important how the surviving neurons adapt to the loss of other neurons and how they succeed to compensate for the neural networks through new connections or increased delivery of neurotransmitters (Enciu et al. 2011). There are well known factors influencing neuroplasticity, such as age, experience and learning, genetic factors, healthy state/comorbidities of the individual, exercise, diet and sleep, age being the most important (Pickersgill et al. 2022). A reduction in neuroplasticity with age, however, might not only be an effect of cells own biological clock. It might be so because we need a set of unchangeable neural networks defining our own selves, our personal identities as individuals.

### Physiological versus pathological neuroplasticity

*Physiological neuroplasticity* refers to the ability of the brain to modify, change and adapt as a result of normal sensory, motor, and cognitive experiences. This type of neuroplasticity is commonly positive, functional, and beneficial, promoting learning and healthy brain development (Morris et al. 1990). Engaging in new learning activities stimulates synaptic plasticity, through complex molecular events, mainly linked to long term potentiation (LTP). LTP is a process where synaptic connections between neurons become stronger with frequent activation and involves glutamatergic signalling through ionotropic α-amino-3-hydroxyl-5-methyl-4-isoxazole-propionate (AMPA) (Diering and Huganir 2018) and N-methyl-D-aspartate (NMDA) (Park et al. 2013) receptors. The resulting calcium influx activates calcium/calmodulin-dependent protein kinase II (CaMKII) – Nicoll and Schulman 2023, protein kinase C (PKC), and mitogen-activated protein kinase (MAPK). With high-rate stimulation of neuronal networks, changes in AMPA receptor trafficking occur, with externalisation of more AMPA receptors on the neuronal membranes, increasing the postsynaptic sensitivity to glutamate and enhancing the synaptic strength (Lisman 2017). Meanwhile, changes in gene expression and protein synthesis take place, making the structural changes possible at the synapse level, with growth of new synaptic connections and strengthening of the existing ones. Over time, sustained LTP leads to permanent changes in neuronal terminal structures, including increased dendritic branching and new synaptic contacts, further stabilizing the enhanced signalling between neurons in the specific activated network (Kuba and Kumamoto 1990). Opposed to LTP, when a neuronal network is not stimulated for a significant period of time, long term depression (LTD) occurs (Connor and Wang 2016) which results in loss of the function of the respective neuronal network (‘*forgetting*’).

Physiological neuroplasticity is subject to a complex regulation. Trophic factors, such as brain-derived neurotrophic factor (BDNF – Fakhoury et al. 2022), neurotransmitters (such as serotonin – Higa et al. 2024 or dopamine – Bech et al. 2023), dedicated genes (such as Nogo-A – Schwab 2010) or even cells (such as microglia – You et al. 2024 and astrocytes—Andrade-Talavera et al. 2023) are able to stimulate or limit the capacity of neurons to make new connections.

We use here the term *pathological neuroplasticity* for changing in connections and rewiring in pathological conditions, such as traumatic brain injury, stroke, neurodegenerative disorders or any other insults or conditions. While this type of plasticity can lead to functional adaptations that help the brain to compensate for damage, it can also result in maladaptive changes that exacerbate dysfunction or manifest as neurological or psychiatric symptoms. A typical example of such a maladaptive neuroplastic response is late-onset vascular epilepsy, where after a stroke the new network formation leads to formation of a seizure focus (Heuts-van Raak et al. 1996). For the subject of this review, the most discussed issue of maladaptive (or aberrant) neuroplasticity is levodopa-induced dyskinesia (Bove et al. 2024). At the other end of this view, the useful compensatory neuroplasticity is represented here by the capacity of neurons in substantia nigra pars compacta to adapt and increase the supply of dopamine in the striatum for a long period of time. It is known that the onset of motor signs of parkinsonism occurs only when the number of nigral neurons decrease to around 30–50% (Lang and Losano 1998, Dauer and Przedborski 2003). There is data suggesting that the surviving nigral neurons do undergo important plastic changes at the level of protein expression, enzymatic transformation of levodopa to dopamine, axonal terminals contacts in the striatum, which succeed to compensate the cellular loss for a period of time (Cheng et al. 2010). However, if we consider the progressive neurodegeneration illustrated by Braak’s pathological stages of PD (Braak et al. 2002), such compensatory neuroplastic events might be predicted for stage IV (transitional stage affecting basal ganglia) and V-VI (cortical stages).

### Basal ganglia pathways and synaptic plasticity

The basal ganglia are involved in a multiplicity of motor and behavioural functions. Most importantly they are implicated in implicit memory formation, motor learning and automation of movement and behaviour (Holly et al. 2024). These functions are heavily dependent on the potential of synaptic plasticity within the basal ganglia circuitry, and hence on the functional organization of cortico-subcortical networks (Del Rey and García-Cabezas 2023).

The classical description of this organization is based on the original model of Alexander and DeLong (DeLong et al. 1984), which has undergone several modifications following clinical observations, neuroimaging data, electrophysiological and neurochemical research in humans as well as in animals. However, one of the basic principles of the original models has prevailed, which is the existence of a pathway promoting movement and action (the “direct” pathway) and a separate inhibitory pathway (the “indirect” pathway). The balance between both pathways is maintained by the release of dopamine in the striatum from the terminals of ventral mesencephalic and nigral neurons. Dopamine exerts opposite effects on both pathways with excitatory effects on D1 receptors in the medium spiny striatal neurons of the direct pathway (dMSN), and inhibitory action on D2 receptors of neurons of the indirect pathway (iMSN) (DeLong and Wichmann 2009). Dopamine release occurs both a phasically and tonically. Phasic release is dependent on reward and creates a critical time window of a few seconds that allows to induce plasticity in corticostriatal dMSN and iMSN synapses related to the preceding action (Andrzejewski et al. 2013).

Although this model allows the prediction of some clinical observations, there are still many gaps, which are only partially filled by subsequent modifications of the model, such as the addition of the hyperdirect pathway, bypassing the striatum and directly delivering cortical information to the subthalamic nucleus (STN), and the connectivity of subcortical nuclei with brainstem reticular and cerebellar nuclei (Doyon and Benali 2005).

#### Corticostriatal pathways

Most of our knowledge on synaptic plasticity in the basal ganglia and its alterations in pathological conditions such as Parkinson’s Disease (PD) refers to corticostriatal connections and striatal interneurons, largely ignoring plastic alternations in other basal ganglia nuclei. The striatum receives massive glutamatergic projections to medium spiny neurons from numerous cortical regions (Gómez-Ocádiz and Silberberg 2023). The main mechanisms of plasticity are different in the direct and indirect pathway (Jin and Costa 2015). In the direct pathway a classical LTP mechanism is predominant; it requires the activation of NMDA-receptors and intracellular kinases, which is promoted by D1 receptor activation in dMSN by dopamine release. In the indirect pathway plasticity is dominated by LTD, which relies heavily on retrograde endocannabinoid signalling (Lovinger, 2010). Activation of metabotropic receptors and the ensuing Ca-influx in iMSN generates the retrograde release of endocannabinoids which lower glutamate release from presynaptic corticostriatal afferents by activation of endocannabinoid receptors. LTD is further promoted by dopaminergic activation of D2receptors. Interneurons in the striatum releasing acetylcholine to muscarinic receptors of medium spiny neurons attenuate synaptic plasticity through the lowering of Ca-influx and kinase activation as well as promoting LTD (Lovinger 2010). The effects of nitric oxide and adenosine further shape the potential of synaptic plasticity in the striatum (Shen et al. 2022, Scarduzio et al. 2022).

Progressive degeneration of dopaminergic terminals in the striatum is a hallmark of PD. The onset of motor symptoms occurs after a loss of some 50–60% of nigrostriatal afferents, which corresponds to a loss of 30% of dopaminergic neurons in substantia nigra (Cheng et al. 2010). A decrease of dopaminergic innervation at dMSN and iMSN leads to a profound reorganization of synaptic plasticity. In the early stages phasic dopamine release will progressively decrease, disturbing context-dependent synaptic plasticity (Madadi Asl et al. 2022). Mechanisms underlying glutamate-induced LTP are no longer supported, affecting mainly dMSN-related plasticity. Due to the loss of D2receptor activation, LTD is affected through a decrease of retrograde endocannabinoid signalling, with a major effect on iMSN and indirect pathway related plasticity. Dopaminergic loss thus contributes not only to hypokinesia as predicted by the model, but also to an impairment of implicit learning and automation of movement and behavior, underlying the major difficulty encountered in the rehabilitation of PD patients (Madadi Asl et al. 2022). As dopaminergic degeneration progresses to a near total loss of striatal dopaminergic terminals there is a progressive loss of the potential for synaptic plasticity, which correlates with the increasing need to rely on external cues and non-automatic behaviour in PD patients, placing additional stress on working memory and cognitive resources (Cousineau et al. 2022).

An important phenomenon that follows a loss of dopaminergic input to MSN in the striatum is the increase of excitability, especially in dMSN, related to a reduction of the inward-rectifying potassium current (Kir), at least in animal models (Li et al. 2024). This increases the excitability of dMSN to incoming stimuli and compromises the formation of a balanced motor output (Li et al. 2024). There are other mechanisms in progressing PD not directly related to dopamine loss that further contribute to the deterioration of corticostriatal plasticity, including the increase of acetylcholine release from activated interneurons and the loss of nitric oxide related “brakes” on glutamatergic activity in iMSN (Shen et al. 2022, Scarduzio et al. 2022).

As levodopa may restore phasic signalling in dMSN in the early stages of PD, motor learning may improve under treatment. At more advanced stages however, where dopaminergic innervation is extremely affected, context-dependent phasic signalling will be lost in both direct and indirect pathways, even under treatment, leading to inappropriate onset of motor activity and impaired motor learning (Albin and Leventhal 2017). It is postulated that levodopa may act as a neurotransmitter itself, or that levodopa conversion to dopamine will occur in serotonergic neurons which are not regulated by the necessary reward-related stimuli (Gantz et al. 2015). The subsequent loss of temporo-spatially coordinated activation of D1 and D2 receptors prevents a structured response to incoming stimuli, leading to random motor activation as reflected in dyskinesia. These considerations explain why the occurrence of levodopa-related dyskinesia requires a degeneration of the dopaminergic system. It also offers an explanation for the anti-dyskinetic effects of NMDA-receptor antagonists, such as amantadine which dampens glutamatergic actions at hyperexcitable dMSN (Duty 2012).

This review of neuroplasticity in corticostriatal connections is far from a final picture. Recent research has indicated that the segregation of direct and indirect pathways may not be as rigid as formerly thought; the existence of collaterals between both pathways has been shown, as well as the existence of D1/D2 heterodimers (Gagnon et al. 2017). These recent findings demonstrating the intertwining of direct and indirect pathways further complicate our understanding of synaptic plasticity in the basal ganglia circuitry.

#### The STN and the hyperdirect pathway

More recent versions of the basal ganglia circuitry have attributed a more central role for the activity of the STN. The STN is a relay nucleus in the indirect pathway of the basal ganglia circuitry, with GABA-ergic connections from the external pallidum (DeLong et al. 1984). In addition, the STN is a direct input nucleus, receiving somatotopically organized glutamatergic inputs from the cortex. The latter is called the hyperdirect pathway (Nambu et al. 2002). The output of the STN is an excitatory glutamatergic projection to the internal pallidum and substantia nigra pars reticulata. The role of the STN in shaping voluntary motor and behavioural responses is undisputed and is illustrated by the observations resulting from lesions and dysfunctions, such as hemiballismus. The STN is thought to be critical in the selection of wanted motor programs and suppressing competing ones (Nambu 2002). This shaping necessitates an early STN excitation by the hyperdirect pathway and a late excitation from the indirect pathway (Polyakova et al. 2020). It is generally assumed that in PD there is a disinhibition of the excitatory STN output, resulting in overactive inhibition of thalamic neurons by the internal pallidum, leading to hypokinesia. This is also assumed to be the rationale for the success of STN stimulation in PD patients.

Recent data suggest that the hyperdirect pathway also undergoes neuroplastic changes. In a parkinsonian mouse model it was shown that the progressive decrease of dopamine in the striatum triggers a subsequent maladaptive excitation/inhibition shift in the STN. This phenomenon seems to be dependent on NMDA receptor signalling in the STN neurons (Chu et al. 2017). With low dopamine levels, there is an increase in NMDA receptors level which triggers an abnormal strengthening of GPe-STN inputs as well (Chu et al. 2015).

In addition, there is evidence for adaptive mechanisms at the level of the synapses between STN and internal pallidum, which are able to modify the impact of the hyperdirect pathway on the basal ganglia output nuclei. These neuroplastic alterations are suggested to depend on LTD induction by retrograde endocannabinoid signalling (Gorodetski et al. 2018), which opens perspectives for pharmacological modulation of these synapses in PD.

### Exercise-driven plasticity of neural networks in PD

At present, symptomatic antiparkinsonian medication allows a relevant motor and functional improvement in PD for many years (Foltynir et al. 2024). In addition to symptomatic drugs, other non-pharmacological approaches have been advised including physical exercise. As Eric Ahlskog already suggested a decade ago, ‘often overlooked (…) is the potential benefit of sustained vigorous exercise on PD progression’ (Ahlskog 2011). Physical exercise symptomatically improves PD (Comella et al. 1994; Kim et al. 2023) and there are clinical and experimental clues to suggest that intensive exercise may change the natural evolution of PD (Ahlskog 2011, 2018; Garcia Ruiz et al. 2022; Tsukita et al. 2022). Finally, physical exercise may reduce the risk of PD and other neurodegenerative diseases (Xu et al. 2010; Belvisi et al. 2020; Sujkowski et al. 2022). In summary, for many, physical exercise might be the first neuroprotective treatment available for PD (Ahlskog 2011, 2018; Garcia Ruiz et al. 2022; Tsukita et al. 2022). The multiple and fascinating mechanisms of exercise include modulation of neuroplasticity.

Physical exercise has been recommended since classic times (Hippocrates and Galen) as a general measure for health (Tipton 2014), but its mechanism has been completely unknown for centuries. At present, some of the potential mechanisms have been studied both in experimental animal models and in patients with neurodegenerative diseases. Several clues suggest that physical exercise may act on many aspects of neuroplasticity (Hirsch et al. 2016; Sacheli et al. 2019; Johansson et al. 2020; Binda et al. 2021; Yu et al. 2023). Neuroplasticity may explain (at least partially) several clinical and experimental observations such as the long-term exercise-related motor benefit, the reduction of brain atrophy, the increased functional connectivity of the anterior putamen, the enhanced dopamine release, and the increased striatal synaptic integrity in animal models (Hirsch et al. 2016; Sacheli et al. 2019; Johansson et al. 2020; Binda et al. 2021; Yu et al. 2023; Marino et al 2023). The exact mechanisms of exercise-related neuroplasticity are unknown, but several relevant findings have been found.

Recently, Marino et al (2023) studied the synaptic mechanisms underlying exercise-induced plastic effects on the striatal synaptic connections. In a very elegant experimental PD animal model, using bilateral intrastriatal injection of α-synuclein (α -Syn) preformed fibrils (PFFs) they observed loss of LTP in striatal spiny projection neurons. After intensive exercise, a lasting rescue of a physiological corticostriatal LTP was observed. If exercise-related neuroplasticity can change the synaptic striatal organization and function, then several molecular mechanisms must be involved. Possibly, the most interesting and testable molecular mechanism includes the release of BDNF (Marino et al 2023; Kowiański et al. 2018; Ruiz-González et al. 2021; Rotondo et al. 2023; de Sousa 2020); suffice is to recall that BDNF is a crucial neurotrophic factor with multiple roles on regulation of neurophysiological processes (Ruiz-González et al. 2021). The BDNF isoforms interact with different types of receptors (including sortolin and p75 neurotrophin receptor) triggering a wide range of signalling cascades (Ruiz-González et al. 2021). BDNF has multiple and overlapping mechanism such as anti-apoptotic activity, regulation of protein synthesis, cytoskeleton development, enhancement of dendritic growth and branching (Marino et al 2023; Kowiański et al. 2018; Ruiz-González et al. 2021; Rotondo et al. 2023; de Sousa 2020). Physical exercise increases plasma BDNF levels in experimental models and individuals with neurodegenerative disorders (Marino et al. 2023; Kowiański et al. 2018; Rotondo et al. 2023) and interestingly, BDNF receptor blockade prevents the beneficial effects of exercise in animal models (Real et al. 2013).

Other potential mechanisms may include elevated expression of anti-inflammatory cytokines, reduced levels of pro-inflammatory cytokines and activated microglia (Svensson et al. 2015). Mitochondrial function has been also investigated. Overexpression of α-Syn results in mitochondrial DNA damage and repressed activation of respiratory chain complex I and IV (Sung et al. 2012). Intensive exercise improves mitochondrial machinery in animal models of PD (Koo et al. 2017), improves mitophagy through the PINK1/Parkin signalling pathway and induces the expression of multiple mitochondrial protein import components such as TOM20, TOM22, and TIM23 (Koo et al. 2017; Li et al. 2023).

In summary, exercise is one the main approaches for the comprehensive treatment of PD and may influence several basic aspects of its pathophysiology (Ahlskog 2011, 2018; Binda et al. 2021; Marino et al. 2023). Exercise acts on several aspects of neuroplasticity and probably can positively change the natural evolution of PD (2,10–15 Ahlskog 2011, 2018; Yoon et al. 2021; Garcia Ruiz et al. 2022; Tsukita et al. 2022).

### Levodopa-induced dyskinesia and aberrant neuroplasticity

Neuroplasticity is central to understanding the pathophysiology of levodopa-induced dyskinesia (LID), a common and debilitating complication in PD therapy in moderate to advanced PD patients. The adaptive capacity of the brain while generally beneficial for recovery from different insults, can unfortunately lead as well to maladaptive changes resulting in clinical complications, such as those observed in LID. Since this subject was extensively reviewed recently (Bove et al. 2024) we will mention here only the main current information regarding LID. The molecular mechanisms underlying LID-associated neuroplasticity involve complex alterations in dopamine receptor signalling pathways. Prolonged levodopa oral (intermittent) treatment leads to heightened sensitivity and responsiveness of D1 receptors on the dMSNs in the striatum (Guigoni et al. 2005). This sensitisation results in the dysregulation of cAMP/PKA signalling pathway and an increase in glutamate neurotransmission, facilitating excessive and uncontrolled motor outputs (Serra et al. 2021). Additionally, the neuroplastic changes in LID are associated with altered expression and function of other neurotransmitter systems, further complicating the synaptic environment and motor outcomes. For instance, alterations in the serotoninergic system, which compensates for dopamine loss, can exacerbate dyskinesia due to its role in modulating striatal output pathways (Cirillo et al. 2024). Probably there are ways to interfere with this aberrant neuroplastic process – a recent review suggests, for instance, that cotreatment with dopamine agonist alters the maladaptive changes (Espa et al. 2023).

### Deep brain stimulation effects on neuroplasticity in PD

Deep brain stimulation (DBS) implies a brain surgical procedure/implant which was developed in 1980s based on the theoretical opportunity of electrical modulation of neurocircuitries in the brain (Benabid et al. 1987). DBS proved valuable for different neurological conditions, such as PD, essential tremor, dystonia, epilepsy and it is under evaluation for few others. Even though an important number of clinical and electrophysiological studies already explored the effects of this intervention, the exact mechanism of DBS action is still unknown, one of the proposed outcomes being the alteration in local neuronal networks neuroplasticity (Kricheldorff et al. 2022). Even the simple insertion of the electrodes, which is known to transitory confer some symptom relief, appears to induce a series of brain tissue reactions, including inflammation, neurotransmitter release and neuroplastic local circuitry alterations, as seen in histological samples in animal models (Hamani et al. 2024). One of the main issues discussed as DBS effect on neuroplasticity is linked to its efficacy in reducing LID. LID is supposed to develop due to aberrant neuroplasticity linked to both degeneration and intermittent dopaminergic stimulation and it was claimed that STN DBS might reverse this phenomenon, since dyskinesias diminished in patients who did not decrease levodopa dose after stimulation as well (Figueiras-Méndez et al. 1999). Using recordings in STN-DBS PD patients, Milosevic and co-authors suggested that enhancement of inhibitory synaptic plasticity with GABA-mediated increasing refractory depression might be a crucial effect of STN-DBS which might explain the alleviation of LID (Milosevic et al. 2018).

Reviewing the general mechanisms of electrical brain stimulation, McIntyre and Anderson postulate that these are multiple and at different levels: ionic level, protein level, cellular level and network level, claiming that DBS might be considered a chemical therapy, since it results in action potential, orthodromic and antidromic propagation of action potentials and finally a very high number of synaptic events (McIntyre and Anderson 2016). Repeated high frequency stimulation is to be expected to result in neuroplasticity activation, as physiologically happen in LTP, for instance, as well. A report suggests that STN stimulation activates antidromically the hyperdirect pathway, the action potentials reaching the cortex and inducing a profound synaptic suppression, with effects on synaptic networks functioning (Anderson et al. 2018).

Since the neurobiological outcomes can be much easier dissected out in animal models, PD murine models exposed to DBS were explored from different experimental perspectives. Helf and collaborators used a rat 6-hydroxydopamine PD model with STN stimulation and showed that after one week, the intervention group had 3.5 more tyrosine-hydroxylase positive neurons in the substantia nigra pars compacta as compared to the control group, suggesting a neurorestorative effect of the high frequency stimulation (Helf et al. 2023).

Beside possible effects on neuroplasticity, in a rat model of PD it was recently shown that DBS is able to significantly increase neurogenesis as well. Beside improving motor function, STN electrical stimulation for 1 week at 130 Hz was able to enhance proliferation of neural stem cells and neuroblast in the subventricular zone and to increase dopaminergic neuronal survival (Wu et al. 2024).

### Biomarkers for neuroplasticity in PD – are we there?

Understanding and identifying biomarkers of neuroplasticity in PD is crucial for developing therapeutic strategies aimed at enhancing neural adaptability, improving clinical symptoms, and ultimately compensating for neurodegeneration. Potential biomarkers of neuroplasticity in PD should encompass a range of molecular, cellular, and imaging indicators that reflect the brain's adaptive changes to specific stimuli and interventions. These include alterations in neurotrophic factors, synaptic proteins, and functional as well as structural brain changes. While studies on factors influencing neuroplasticity have exponentially increased in the past two decades, biomarkers to assess, quantify and predict neuroplasticity have not yet been standardised and validated. This is partly due to the heterogeneity of changes that can reflect adaptive brain processes, such as synaptic plasticity. For instance, in a recent systematic review and meta-analysis by Hortobàgyi et al. (2022) assessing the impact of aerobic and resistance training intensity on neuroplasticity in health and disease, 74 markers of brain activation, 94 markers of brain structure, and 76 markers of neurochemicals were extracted from 50 selected studies (Hortobágyi et al. 2022).

In the context of PD, neuroplasticity biomarkers have been used mainly in studies investigating the effects of non-pharmacological interventions. For the purpose of this review, these can be classified as biofluid-based biomarkers, imaging biomarkers or biomarkers derived from other techniques.

### Biofluid-based biomarkers

Most clinical studies assessing the effects on neuroplasticity of specific interventions, such as physical activity and rehabilitation-based programs, have assessed blood levels of neurotrophins such as brain-derived neurotrophic factor (BDNF) and insulin-like growth factor-1 (IGF-1), among others (Rotondo et al. 2023). The greatest evidence in PD has been gathered so far for BDNF, which is involved in neuronal development, synaptic plasticity, LTP, and is crucial for neurogenesis and synaptic modulation (Park & Poo 2013). Serum BDNF levels have been shown to raise following exercise therapy and seem to be associated with improved motor symptoms, according to the results of two recent meta-analyses (Kaagman et al. 2024; Paterno et al. 2024). However, contrasting results have also been reported and it is not clear which type of physical activity or rehabilitation training could be more effective in increasing blood BDNF levels (Rotondo et al. 2023).

Similarly to BDNF, also IGF-1 could be seen as a potential biomarker for neuroplasticity in PD, given the possible contribution of impaired insulin/IGF-1 signalling in PD pathophysiology, and the increased IGF-1 levels in serum and CSF of PD patients (Godau et al. 2010). However, no significant changes were reported in two studies assessing changes in IGF-1 levels following physical interventions, possibly due to the small sample sizes (Rotondo et al. 2023; Soke et al. 2021; Szymura et al. 2020).

There is also some evidence for a decrease in inflammatory cytokines, such as serum tumor necrosis factor-alpha (TNF-alpha), and basal serum soluble vascular adhesion molecule 1 (sVCAM-1) in response to moderate-intensity interval training in PD, suggesting that reduced inflammation may contribute to increased neuroplasticity observed with physical training (Zoladz et al. 2014).

### Imaging biomarkers

Functional and structural brain changes in response to physical exercise (aerobic, resistance, endurance, etc.) and/or virtual setting (virtual reality and motor imagery) in PD have been explored by several studies, which has been summarised by Baglio et al. in a recent systematic review (Baglio et al. 2022). Among the selected studies, the majority used functional MRI (fMRI), either task-based or resting state, with one combining it with arterial spin labelling (ASL) technique (Baglio et al. 2022). Only one structural MRI study was selected (Sehm et al. 2014). Overall, the effectiveness of moderate aerobic exercise seemed to be based on improved functionality within the cortico-thalamic-striatal pathways, while goal-directed exercise seemed to act on supplementary circuitries supporting the sensorimotor integration and reinforcing the coupling of premotor areas, often affected in PD since the early stages (Baglio et al. 2022; Sehm et al. 2014; Silva-Batista et al. 2020). Interestingly, two studies assessing the effects of cognitively-enhanced exercise programs, showed that the observed clinical improvements in cognitive and motor symptoms –particularly gait – were associated with changes in functional connectivity mainly across the frontoparietal network (Agosta et al. 2017; Maidan et al. 2017).

In terms of molecular imaging, a small pilot study using PET imaging with [18F]fallypride in individuals with early-stage PD showed that intensive exercise led to an increase in binding potential in the dopamine D2 receptor, suggesting exercise-derived neuroplasticity in dopaminergic signalling (Fisher et al. 2013).

### Other biomarkers

Few studies have attempted to quantify neuroplasticity processes following interventions in PD using alternative methods. For example, Fischer et al. observed a post-exercise increase in corticomotor excitability in response to single-pulse transcranial magnetic stimulation (TMS) in a small randomised controlled trial (Fisher et al. 2008).

Interestingly, Mougeot et al. investigated potential markers of exercise-induced neuroplasticity in the oral cavity, based on the hypothesis that exercise can affect the activity of submandibular glands through noradrenergic inputs (Mougeot et al. 2016).

Overall, the current evidence for reliable biomarkers of neuroplasticity is limited by small sample sizes and heterogeneous definitions of neuroplasticity. The correlation with clinical improvements (motor and/or non-motor) is yet to be clarified for most of the proposed biomarkers. Additionally, neuroplasticity in PD has been investigated mostly for exercise-based interventions, therefore limiting the use of these markers in relation to other types of interventions, including pharmacological.

## Conclusions

Our brain has an extraordinary capacity to adapt not only to external conditions but also to internal problems, such as insults and pathology. Using specific endogenous defence mechanisms, the brain is able to reconnect and change the neuronal milieu, protein expression and signalling in specific areas and networks. In PD, a first effect of cell loss is an increase in efficacy of surviving neurons, which compensate the pathology for a period of time. Unfortunately, there are also negative effects in this adaptation to the neurodegenerative background, resulting in complications such as dyskinesia. Further studies should focus on possible therapeutical targets to increase the compensatory effects and limit the maladaptive effects of neuroplasticity in PD. In any case, in the search for disease-modifying treatment, we must take into account neuroplasticity, among other factors.

## Data Availability

Not applicable.

## Abbreviations

α -Syn: α-Synuclein
AMPA: α-Amino-3-hydroxyl-5-methyl-4-isoxazole-propionate
ASL: Arterial spin labelling
BDNF: Brain-derived neurotrophic factor
cAMP: Cyclic adenosine monophosphate
CaMKII: Calcium/calmodulin-dependent protein kinase II
CSF: Cerebrospinal fluid
DBS: Deep brain stimulation
dMSN: Medium spiny striatal neurons of the direct pathway
DNA: Deoxyribonucleic acid
GPe: Globus pallidus external part
IGF-1: Insulin-like growth factor-1
iMSN: Medium spiny striatal neurons of the indirect pathway
Kir: Inward-rectifying potassium current
LID: Levodopa-induced dyskinesia
LTD: Long term depression
LTP: Long term potentiation
MAPK: Mitogen-activated protein kinase
MRI: Magnetic resonance imaging
NMDA: N-methyl-D-aspartate
PD: Parkinson’s disease
PET: Positron emission tomography
PFFs: Preformed fibrils
PINK1: PTEN-induced kinase 1
PKA: Protein kinase A
PKC: Protein kinase C
STN: Subthalamic nucleus
sVCAM-1: Serum soluble vascular adhesion molecule 1
TOM: Translocase of the outer membrane
TIM23: Mitochondrial import inner membrane translocase subunit TIM23
TMS: Transcranial magnetic stimulation
TNF-alpha: Tumor necrosis factor-alpha
